# Generating a focused view of disease ontology cancer terms for pan-cancer data integration and analysis

**DOI:** 10.1093/database/bav032

**Published:** 2015-04-04

**Authors:** Tsung-Jung Wu, Lynn M. Schriml, Qing-Rong Chen, Maureen Colbert, Daniel J. Crichton, Richard Finney, Ying Hu, Warren A. Kibbe, Heather Kincaid, Daoud Meerzaman, Elvira Mitraka, Yang Pan, Krista M. Smith, Sudhir Srivastava, Sari Ward, Cheng Yan, Raja Mazumder

**Affiliations:** ^1^Department of Biochemistry and Molecular Medicine, George Washington University, Washington, DC 20037, USA, ^2^Institute for Genome Sciences, University of Maryland School of Medicine, Baltimore, MD 21201, USA, ^3^Center for Bioinformatics and Information Technology, National Cancer Institute, 9609 Medical Center Drive, Rockville, MD 20892-9760, USA, ^4^NASA Jet Propulsion Laboratory, Pasadena, CA, USA, ^5^Division of Cancer Prevention, National Cancer Institute, 9609 Medical Center Drive, Rockville, MD 20892-9760, USA, ^6^Wellcome Trust Sanger Institute, Cambridge, UK and ^7^McCormick Genomic and Proteomic Center, George Washington University, Washington, DC 20037, USA

## Abstract

Bio-ontologies provide terminologies for the scientific community to describe biomedical entities in a standardized manner. There are multiple initiatives that are developing biomedical terminologies for the purpose of providing better annotation, data integration and mining capabilities. Terminology resources devised for multiple purposes inherently diverge in content and structure. A major issue of biomedical data integration is the development of overlapping terms, ambiguous classifications and inconsistencies represented across databases and publications. The disease ontology (DO) was developed over the past decade to address data integration, standardization and annotation issues for human disease data. We have established a DO cancer project to be a focused view of cancer terms within the DO. The DO cancer project mapped 386 cancer terms from the Catalogue of Somatic Mutations in Cancer (COSMIC), The Cancer Genome Atlas (TCGA), International Cancer Genome Consortium, Therapeutically Applicable Research to Generate Effective Treatments, Integrative Oncogenomics and the Early Detection Research Network into a cohesive set of 187 DO terms represented by 63 top-level DO cancer terms. For example, the COSMIC term ‘kidney, NS, carcinoma, clear_cell_renal_cell_carcinoma’ and TCGA term ‘Kidney renal clear cell carcinoma’ were both grouped to the term ‘Disease Ontology Identification (DOID):4467 / renal clear cell carcinoma’ which was mapped to the TopNodes_DOcancerslim term ‘DOID:263 / kidney cancer’. Mapping of diverse cancer terms to DO and the use of top level terms (DO slims) will enable pan-cancer analysis across datasets generated from any of the cancer term sources where pan-cancer means including or relating to all or multiple types of cancer. The terms can be browsed from the DO web site (http://www.disease-ontology.org) and downloaded from the DO’s Apache Subversion or GitHub repositories.

**Database URL:** http://www.disease-ontology.org

## Introduction

Cancer is among the leading causes of deaths worldwide and accounts for 8.2 million deaths annually (1). The term cancer describes a group of diseases in which cells develop abnormal abilities to grow rapidly without limitation, invade adjoining tissues and metastasize to other parts of the body through the lymph and blood systems (2, 3). In general, cancers arise from one abnormal cell that goes through multiple transformations from normal to malignant states. Normal, non-cancerous cells grow and divide into additional cells and undergo a limited number of division cycles in a controlled manner (4, 5). When cells are damaged or old, they undergo the processes of apoptosis, programmed cell death or necrosis (6, 7). However, interactions between genetic factors and physical, chemical and biological carcinogens can disrupt this order causing damage and alteration to DNA structures (8, 9). As a result of these mutations, cells proliferate without restraint, leading to tumor formation and variety of cancers (4).

It is suggested that the word cancer comes from the Greek word karkinos which was used by Hippocrates (460–370 BC) (10). Since then different types of cancer have been recognized based on their anatomical origin, disease potential, histopathological characteristics and molecular and genetic makeup (11–13). In research, cancer types are often referred to by their primary organ sites, such as skin, liver, breast and prostate cancers. However, since organs may consist of two or more types of cells, using the primary origin site as classification criteria may not provide the most accurate and precise definition of tumors. Another option that is widely used is to sort cancers based on six primary histological categories: carcinoma, sarcoma, myeloma, leukemia, lymphoma and mixed types. Tumors can also be classified by several different systems, including grading (14) and staging systems (15). The tumor grading system describes tumors by comparing abnormal cells or tissues with normal cells or tissues under microscopic observation. For this system, tumors in different organs or tissues are graded by specific classification criteria to determine tumor grade. Conversely, staging systems describe cancer severity based on the size of the original tumor and whether the cancer has spread throughout the body (16). One of the most widely used staging systems, the TNM system, uses primary tumor size (T), affected nearby lymph nodes (N) and the presence of metastasis (M) (either locally or spread to distant parts of the body) to classify tumors. Each category in this system combines numeric and alphabetic parameters to categorize tumor severity. However, the TNM stage system can only be applied to certain tumor types. Some types of tumors, such as brain tumors, do not have corresponding categories in the staging system and must be classified by grading systems (15).

With genomic and molecular data becoming available for the majority of cancer types, there is a marked increase of research efforts to compare and contrast mutation, expression and other genomic characteristics across multiple cancers (17–24). The majority of these studies use data from just one database or resource: hence, there is usually no ambiguity of the cancer terms under consideration. Studies that attempt to integrate data from multiple databases and publications present challenges to the mapping of terms and data associated with these terms across different resources because database providers or authors may choose to use different terms with the same meaning or more granular terms to describe specific datasets. This type of inconsistency can result in significant barriers for users to integrate and analyse data from multiple sources. There is an urgent need to develop a comprehensive ontology of cancer terms which then can be used to generate a cohesive subset of cancer terms that represent the diversity of cancer types across databases that can be utilized for pan-cancer analysis (17, 25) where pan-cancer means including or relating to all or multiple types of cancer. Ontologies provide controlled terminologies for the scientific community to describe biomedical entities in a consistent and standardized manner (26). A cancer ontology slim represents a subset of specific cancer-related ontology terms that can be used to perform meta-analysis across multiple cancer types to identify driver genes and other molecular determinants (25).

To promote international unity in the collection, processing, classification and presentation of disease-related data, the World Health Organization (WHO) has published the International Classification of Diseases (ICD) to serve as a medical code (27). In addition to WHO, several initiatives have been developed to define healthcare terminology and consistency in terms for electronic medical records, including Systematized Nomenclature of Medicine–Clinical Terms (SNOMED CT) (28), Medical Subject Headings (MeSH) (29), Unified Medical Language System (UMLS) (30), National Cancer Institute Thesaurus (NCIt) and Ontology, ICD-9 and ICD-10 (27, 31). Each of these initiatives focuses on different needs and requirements of the healthcare community. For example, SNOMED CT (28), maintained by the International Health Terminology Standards Development Organization, is the most comprehensive, multilingual clinical healthcare terminology in the world. The major aim of MeSH is to index journal articles from the MEDLINE/PubMed database. UMLS serves as a tool that aids in enhancing and developing electronic health records. However, UMLS emphasizes the creation of more effective and interoperable biomedical information systems and services, which includes distributing key terminology, classification coding standards and associated resources. Meanwhile, ICD focuses on recording causes of death and disease.

Many organizations and multiple terminologies and bio-ontologies have been created for the purpose of providing better annotation. However, a major issue that persists in the field of bio-ontology is dealing with terms that are represented (and overlapping) in several ontologies (26). For example, although many clinical or medical terminologies have been created, cancer definitions often overlap and it is not clear which terminology should be used. For example, MeSH considers the term ‘prostate cancer’ as a prostatic neoplasm that is related to four different tree numbers (C04.588.945.440.770, C12.294.260.750, C12.294.565.625 and C12.758.409.750) and lists 17 similar but not identical entry terms. In SNOMED CT, several concept ID numbers and names [126906006: Neoplasm of prostate (disorder), 254900004: Carcinoma of prostate (disorder), 399068003: Malignant tumor of prostate (disorder) and 93974005: Primary malignant neoplasm of prostate (disorder)] can be found. And, each ‘Name’ includes similar but not the exact the same description of prostate cancer. Granting all controlled vocabulary projects attempt to offer the most accurate and granular information possible, too many search results can be difficult and overwhelming for a general user.

Although there are several methods for classifying cancers as described earlier, the systems are not standardized or cohesive since they have been devised for multiple purposes. Over the past decade, the number of researchers interested in biomedical ontologies has rapidly increased, leading to an increase in the production of these scientific ontologies (32). The gene ontology (GO) Consortium is an example of a widely used ontology established to develop a consistent terminology of gene product attributes across databases (33). In addition to establishing ontologies for biological processes, cellular components and molecular functions for gene products, the GO Consortium developed a GO slim reduced subset of the GO terms. The GO Slim subset provides users with a summary overview of the entire GO without specific granular details of children terms and is used widely for GO enrichment analysis (34). The disease ontology (DO) is an example of ontology established to develop consistent classification of human disease across clinical disease vocabularies and biomedical databases (32) and just like GO slims it is possible to generate DO slims made of top level nodes that can provide summary overview of specific diseases representing granular and highly specific terms.

This article describes the DO cancer project that involves mapping of diverse cancer terms from multiple sources [Catalogue of Somatic Mutations in Cancer (COSMIC) (35), The Cancer Genome Atlas (TCGA) (36), International Cancer Genome Consortium (ICGC) (37), Therapeutically Applicable Research to Generate Effective Treatments (TARGET) (https://ocg.cancer.gov/programs/target), Integrative Oncogenomics (IntOGen) (38) and Early Detection Research Network (EDRN) (39)] to DO terms. A set of 63 upper level DO terms (TopNodes_DOcancerslim) representing the corresponding types of cancer (e.g. breast cancer, lung cancer, brain cancer) was generated, which can be used to integrate and evaluate pan-cancer data from multiple sources. For example, synonymous cancer terms from different databases mapped to specific DO terms can be used to collapse hundreds of terms across many databases into a few top-level cancer types that can then be used to identify mutations or expressions across multiple cancers (17, 40). Therefore, the DO cancer project attempts to provide precise mapping between all of the cancer terms from multiple cancer databases and helps synchronize inconsistent terms across data sites and connect these terms to Online Mendelian Inheritance in Man (OMIM), NCIt, UMLS, MeSH, SNOMED_CT and ICD.

## The disease ontology

The DO is a hierarchical structured classification system of common and rare human diseases (41). DO links and integrates different terminologies, vocabularies and identification numbers of diseases into one single term and identification number. DO builds a clear hierarchical and structural relationship between disease terms and concepts, and it integrates MeSH, OMIM, ICD-9-CM, ICD-10, NCI thesaurus, Orphanet, UMLS and SNOMED CT terms and IDs together into the DO cross-reference section. DO’s classification of cancer terms (disease of cellular proliferation) includes three subtypes: benign neoplasm, pre-malignant neoplasm and cancer. The cancer branch is further subdivided into cell type cancer and organ system cancer. DO enable users to make connections of concepts between cancer and related diseases.

## Mapping of cancer terms to DO

Each of the cancer mutation databases in the study developed their own cancer type nomenclature system to serve their distinct usage requirements and therefore display differences in cancer terms. This creates a major challenge in mapping heterogeneous cancer terms from the multiple resources. In the COSMIC dataset, four descriptions are used to indicate the site of mutation: primary site, site subtype, histology and histology subtype. ICGC used terms similar (but not necessarily identical) to cancer categories defined by TCGA. For example, at the time of writing of this manuscript we noticed that TCGA used the term ‘liver hepatocellular carcinoma’, while in ICGC a Japanese team used the term ‘liver cancer’ and another French team used the term ‘liver tumor’. Another group, IntOGen, downloaded and analysed TCGA datasets with their own algorithms to retrieve pan-cancer variations. However, IntOGen categorized the cancer origin organ level terms, such as liver, prostate and stomach. EDRN, an NCI funded cancer biomarker research organization, contains a cancer classification system that applies organ level terms, sub-organ level terms and pathology level categories to specific cancer types. Thus, a variety of different data sources contained several different cancer descriptions.

The first step was to identify the breadth of terms represented across cancer term sources including COSMIC, TCGA, ICGC, TARGET, IntOGen and EDRN. BioMuta, a non-synonymous single-nucleotide variation (nsSNV) pan-cancer analysis database that correlates and integrates cancer variations from multiple sources, initiated this effort by identifying and integrating terms from several of these databases into DO and TCGA cancer terms (25). The most recent version of mapping contains a set of 386 terms mapped to DO cancer terms. Of the 386 cancer terms, 287 terms were mapped from COSMIC, 24 terms from TCGA, 23 terms from ICGC, 9 terms from TARGET, 12 terms from IntOGen and 31 terms from EDRN. These ‘child nodes’ in DO were then mapped to a common set of ‘parent nodes’ (63 TOPNodes_DOcancerslim) to identify the most appropriate mapping for the diverse set of cancer terms (see Supplementary Table S1). These top-level terms represent 60 COSMIC, 26 EDRN, 21 TCGA, 12 IntOGen, 16 ICGC and 4 TARGET terms.

## Materials and methods

All cancer terms were retrieved from the source database websites in September and October 2014.

### Source database description

*COSMIC*: The COSMIC database contains mutation data and information related to human cancer (42). To provide a precise description of cancer origin of the data, COSMIC combines histology and tissue ontology. Overall, four columns of information is used to define the origin of each mutation (primary site, site subtype, histology and histology subtype). This definition method displays a hierarchical structure similar to DO.

*TCGA:* One of the largest cancer databases maintained by NCI is TCGA, which includes clinical information, genomic characterization data and tumor genome sequence analyses (18). At the time of writing of this manuscript, 11 major grouped categories containing 31 cancer tissue collections was the focus of TCGA group.

*ICGC*: ICGC coordinates research projects around the world encompassing 74 projects and more than 50 different tumor types (43). Through the coordination of these research projects, ICGC has developed a comprehensive catalogue of genomic abnormalities across cancer types (43). Although ICGC cancer terms are mostly consistent with the TCGA nomenclature system, projects initiated by different countries have some minor differences (e.g. Renal Cancer—CN and Renal Cell Cancer—EU/FR).

*TARGET*: TARGET is an initiative managed by NCI to determine the genetic changes driving pediatric cancers (44). TARGET’s mission is to identify biomarkers allowing for the development of better treatment for childhood cancers. Primary data sources for TARGET are collected from multiple institutes and labs within USA. Currently, the TARGET data matrix page contains five major types of cancer which includes 9 datasets which can be summarized into 7 diseases.

*IntOGen*: The IntOGen Browser contains mutation, gene and pathway data across tumor types (38). A major repository of mutation results detected by the IntOGen pipeline is derived from TCGA raw data.

*EDRN*: EDRN is an infrastructure funded through the NCI for supporting collaborative research on molecular, genetic and other biomarkers in early cancer detection and risk assessment (45). The EDRN model is composed of five major components: Biomarker Development Laboratories, Biomarker Reference Laboratories, Clinical Validation Centers, Data Management and Coordinating Center and Informatics Center. The EDRN common data elements cover the following areas: pre-clinical cancer biomarkers, study management, specimens, instrument data (including proteomics, genomic data), publications and investigators that integrate the EDRN into a semantic knowledge system.

## Results and discussion

### Manual mapping of cancer terms

**Figure 1.**
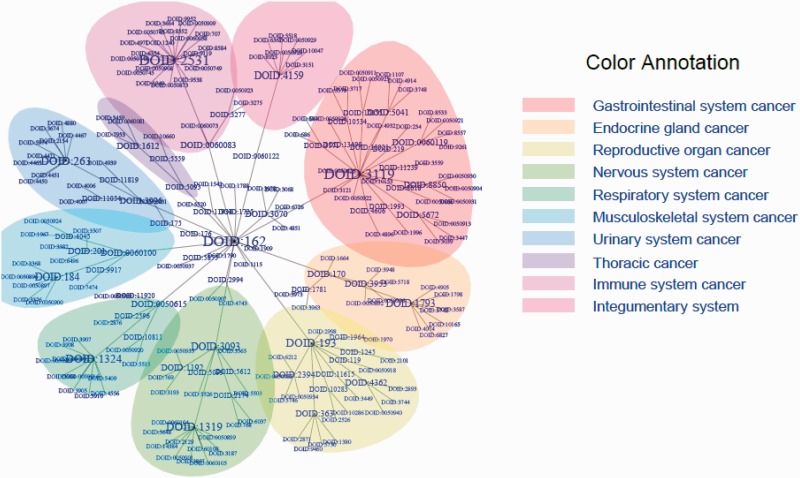
DO cancer tree plot presenting the hierarchical tree structures of the system. The summarized terms (DOIDs, level 1), TopNodes_DOcancerslim (DOIDs, level 2) and child terms (DOIDs, level 3) are included in the tree with DOID: 162 / Cancer as the root. In the case that the same term is used in more than one level, only the highest level is plotted. The branch of the summarized term with more than five nodes is colored as shown. The top-level terms and child terms are available in the Supplementary Table S1. The summarized terms are derived from the level under cell type cancer and organ system cancer of DOID 162 / cancer in DO.

**Figure 2.**
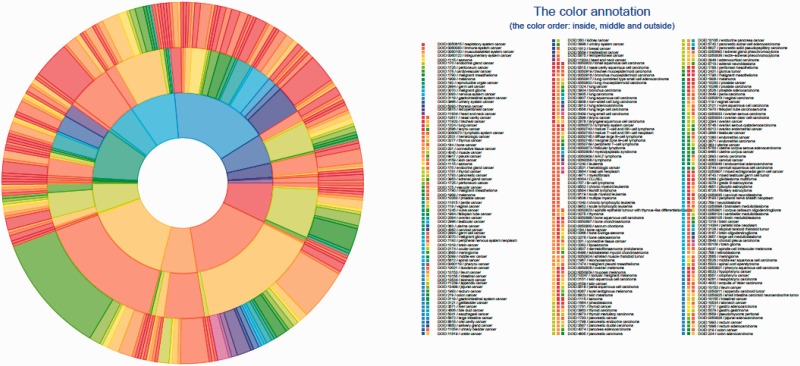
DO cancer Circos plot showing the hierarchical structure of the system. All mapped subsumed terms (the innermost layer), TopNodes_DOcancerslim level terms (the middle layer) and child terms (the outermost layer) are plotted with the full DOIDs/terms listed. The top-level terms and child terms are available in the Supplementary Table S1. The summarized terms are derived from the level under cell type cancer and organ system cancer of DOID / cancer in DO.

The list of 386 cancer terms derived from all of the data sources mentioned earlier were mapped to terms within DO. The manual process of identifying the type of cancer term involved investigation of each cancer term to identify the current classification of each type of cancer from authoritative resources including primary publications, the NCI Dictionary of Cancer terms, NCI cancer topics and WHO classification. This process involved identifying the proper nomenclature for each term and developing a DO definition to describe the disease etiology. Each of these resources were then included as references for the term and definition provenance in DO. The four primary steps to map cancer terms from different data sites to DO terms were as follows: Step 1 involved identifying the name of the cancer term from the data source. For example, central_nervous_system, basal_ganglia, glioma, astrocytoma_Grade_IV, from COSMIC was grade IV astrocytoma. Step 2 involved identifying the current nomenclature for this disease as disease terms change over time. In this example, grade IV astrocytoma mapped to glioblastoma multiforme. Next, in Step 3 we identified if the cancer term existed in DO or if the term could be mapped to a synonym of a DO term or if the term was a novel term and should be added to DO. In this example, glioblastoma multiforme already existed in DO as Disease Ontology Identification (DOID):3068. Step 4 involved investigating the most appropriate definition of the term, and defining all parental terms linked to this primary term. Each term was mapped within DO to the most appropriate cell type or organ system cancer node. These steps resulted in 43 new terms being added to DO and the addition of definitions and references to 63 DO parent terms and 187 DO child node terms. This DO cancer hierarchical structure is presented in Figures 1 and 2 using tree and Circos plots (46). The generated hierarchy represents a cohesive set of DO terms that enables cancer terms to be mapped across cancer resources. Table 1 contains the terms listed in TOPNodes_DOcancerslim.obo file along with their number of ‘Children Nodes’ and ‘Source’ databases.

**Table 1.** DO TopNodes_DOcancerslim terms (detailed mapping is available in Supplementary Table S1)

DOID	DO cancer-slim	Children node	Source
DOID:2531	Hematologic cancer	20	COSMIC,EDRN,ICGC,IntOGen,TARGET,TCGA
DOID:1319	Brain cancer	11	COSMIC,EDRN,ICGC,IntOGen,TCGA
DOID:1324	Lung cancer	11	COSMIC,EDRN,ICGC,IntOGen,TCGA
DOID:263	Kidney cancer	10	COSMIC,EDRN,ICGC,IntOGen,TARGET,TCGA
DOID:1793	Pancreatic cancer	8	COSMIC,EDRN,ICGC,IntOGen,TCGA
DOID:4159	Skin cancer	8	COSMIC,EDRN,TCGA
DOID:184	Bone cancer	6	COSMIC,EDRN,TARGET
DOID:0060119	Pharynx cancer	5	COSMIC,EDRN,IntOGen
DOID:2394	Ovarian cancer	5	COSMIC,EDRN,ICGC,IntOGen,TCGA
DOID:1612	Breast cancer	4	COSMIC,EDRN,ICGC,IntOGen,TCGA
DOID:201	Connective tissue cancer	4	COSMIC,ICGC
DOID:3070	Malignant glioma	4	COSMIC,TCGA
DOID:363	Uterine cancer	4	COSMIC,EDRN,IntOGen,TCGA
DOID:3953	Adrenal gland cancer	4	COSMIC,TCGA
DOID:5041	Esophageal cancer	4	COSMIC,EDRN,ICGC,TCGA
DOID:8850	Salivary gland cancer	4	COSMIC
DOID:10155	Intestinal cancer	3	COSMIC
DOID:10283	Prostate cancer	3	COSMIC,EDRN,ICGC,TCGA
DOID:10534	Stomach cancer	3	COSMIC,EDRN,ICGC,IntOGen,TCGA
DOID:11054	Urinary bladder cancer	3	COSMIC,EDRN,ICGC,IntOGen,TCGA
DOID:1192	Peripheral nervous system neoplasm	3	COSMIC,TARGET
DOID:1781	Thyroid cancer	3	COSMIC,EDRN,ICGC,TCGA
DOID:3571	Liver cancer	3	COSMIC,EDRN,ICGC,TCGA
DOID:4362	Cervical cancer	3	COSMIC,EDRN,ICGC,TCGA
DOID:5672	Large intestine cancer	3	COSMIC
DOID:119	Vaginal cancer	2	COSMIC,EDRN
DOID:11934	Head and neck cancer	2	COSMIC,TCGA
DOID:1993	Rectum cancer	2	COSMIC,EDRN,TCGA
DOID:2174	Ocular cancer	2	COSMIC
DOID:219	Colon cancer	2	COSMIC,EDRN,IntOGen,TCGA
DOID:2596	Larynx cancer	2	COSMIC,EDRN
DOID:2994	Germ cell cancer	2	COSMIC
DOID:3119	Gastrointestinal system cancer	2	COSMIC
DOID:3277	Thymus cancer	2	COSMIC
DOID:4045	Muscle cancer	2	COSMIC
DOID:8618	Oral cavity cancer	2	COSMIC,EDRN,ICGC
DOID:0060073	Lymphatic system cancer	1	COSMIC
DOID:10021	Duodenum cancer	1	COSMIC
DOID:10153	Ileum cancer	1	COSMIC
DOID:10811	Nasal cavity cancer	1	COSMIC
DOID:1115	Sarcoma	1	COSMIC
DOID:11239	Appendix cancer	1	COSMIC
DOID:11615	Penile cancer	1	COSMIC
DOID:11819	Ureter cancer	1	COSMIC
DOID:11920	Tracheal cancer	1	COSMIC
DOID:1245	Vulva cancer	1	COSMIC
DOID:13499	Jejunal cancer	1	COSMIC
DOID:170	Endocrine gland cancer	1	COSMIC
DOID:1725	Peritoneum cancer	1	COSMIC
DOID:175	Vascular cancer	1	COSMIC
DOID:1790	Malignant mesothelioma	1	EDRN
DOID:1909	Melanoma	1	COSMIC
DOID:1964	Fallopian tube cancer	1	COSMIC
DOID:2998	Testicular cancer	1	EDRN
DOID:3121	Gallbladder cancer	1	EDRN
DOID:3565	Meningioma	1	COSMIC
DOID:3996	Urinary system cancer	1	COSMIC
DOID:4606	Bile duct cancer	1	COSMIC
DOID:5099	Middle ear cancer	1	COSMIC
DOID:5559	Mediastinal cancer	1	COSMIC
DOID:5612	Spinal cancer	1	COSMIC
DOID:5875	Retroperitoneal cancer	1	COSMIC
DOID:9917	Pleural cancer	1	COSMIC

The cross-reference section of DO provides synonymous IDs to each DO term from other clinical vocabularies including the NCI thesaurus (NCIt). Utilizing the DO to NCIt ID mappings enabled highly accurate mapping between COSMIC and DO. Similar to COSMIC, most TCGA terms matched perfectly with DO. Most of the terms in TCGA described a single cancer type or single tissue, and DO contained an equivalent term. However, for terms those were too broad or included too many cancer types, such as head and neck squamous cell carcinoma, our group generated new terms in DO to ensure the availability of a matched term. For terms in ICGC that followed the TCGA nomenclature system, the TCGA cancer term mappings to DO was used. For terms in ICGC that did not follow the TCGA nomenclature system, a perfect match or higher node to represent its meaning was adopted. For TARGET, nine projects which included eight disease terms were mapped to DO terms. For example, rhabdoid tumor (RT) was one of the kidney tumor datasets on the TARGET data matrix. The synonym description of kidney rhabdoid cancer in DO clearly stated it was a ‘RELATED’ synonym to kidney rhabdoid tumor. Therefore, we mapped RT to kidney rhabdoid tumor, which provided a match between an adult and childhood disease. IntOGen used organ-level terms as the default representation of original cancer sites. Hence, those terms had a direct translation and match to DO organ system cancer terms. However, there were three exceptions: hematopoietic and reticuloendothelial systems, lung and bronchus, and liver and hepatic bile ducts, which were each represented as different cancer types. For these three types, we decided to move to the adjacent higher-level node to include both types of cancer. EDRN used organ-level terms to describe cancer origin with a few exceptions. Organ-level mapping terms had a direct translation and perfect match to DO organ system cancer terms. For special cases such as ‘skin (melanoma, no basal or squamous)’, we mapped the term to skin melanoma instead of skin cancer.

**Figure 3.**
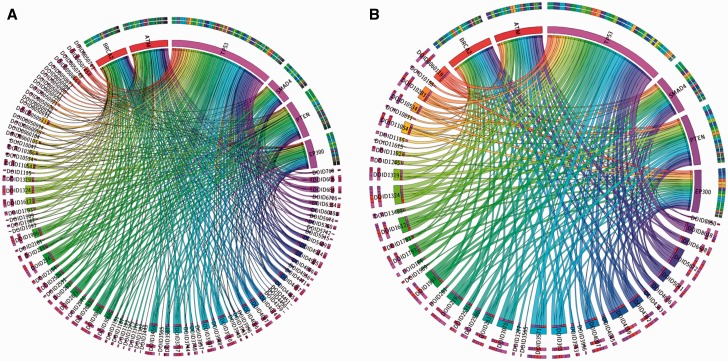
An example showing pan-cancer view of gene mutations mapped to DO cancer terms. **A.** Six oncogenes were mapped to 110 DO terms. The bandwidth represents the number of unique SNVs found in that gene in different cancer types. **B.** TopNodes_DOcancerslim display of the same analysis which shows 46 cancer terms associated with mutations found in six oncogenes. Overall, panel B displays a clearer view and the summarization enables large-scale analysis on an entire set of oncogenes or tumor suppressors across multiple cancer types. DOID terms are available in Table 1. HGNC gene symbols are used to represent the cancer genes.

Figure 3 displays a pan-cancer view of gene mutations mapped to DO cancer terms. In this figure, 6 oncogenes were selected from 13 significantly mutated genes based on our previous pan-cancer analysis (17). The oncogene categories were provided by Vogelstein *et al*. (47) in Cancer Genome Landscape study (Supplementary Table S1). Figure 3A displays 6 oncogenes mapped to 110 DO terms. In this figure, the bandwidth represents the number of unique mutations found in that gene labeled with that cancer type. Figure 3B displays the application of the TopNodes_DOcancerslim which shows 46 cancer terms associated with mutations found in six oncogenes and therefore provides a bird’s eye view of the mapping. Overall, Figure 3B displays a clearer view and the summarization enables large-scale analysis on an entire set of oncogenes or tumor suppressors across multiple cancer types.

## Usage and utility

The DO is available from several websites, in The Open Biological and Biomedical Ontologies (OBO) and Web Ontology Language (OWL) format. The two DO slim files: DO_cancer_slim.obo and TopNodes_DOcancerslim.obo are contained within the HumanDO.obo file, since they are a subset of the ontology itself (http://sourceforge.net/p/diseaseontology/code/HEAD/tree/trunk/HumanDO.obo) and can also be downloaded from DO’s Sourceforge repository or DO’s Github site, https://github.com/obophenotype/human-dis ease-ontology, in the src/ontology folder. Users upon downloading data from any of the cancer genomics databases supported by this project can replace the source terms with DOIDs and DO cancer terms prior to analysis of mutation or expression data from these source databases. Users can also request for new terms to be added to DO if mapping for any term is missing. The entire DO can be viewed and queried at http://www.disease-ontology.org, European Bioinformatics Institute's Ontology Lookup Service (http://www.ebi.ac.uk/ontology-lookup), the OBO Foundry (http://www.obofoundry.org/cgi-bin/detail.cgi?id=disease_ontology) the National Center for Biomedical Ontology BioPortal (http://purl.bioontology.org/ontology/DOID) and OntoBee (http://www.ontobee.org/browser/index.php?o=DOID). DO’s OBO and OWL files can be downloaded from http://www.berkeleybop.org/ontologies/doid.obo or http://purl.obolibrary.org/obo/doid.owl.

This project addresses several challenges involved in integrating data from multiple sources. First, databases (except COSMIC) use similar terms [‘Esophageal Cancer’ in ICGC, ‘Esophageal carcinoma (ESCA)’ in TCGA and ‘esophagus (cancer)’ in EDRN] to indicate the organ source, yet these terms are distinct from one another and therefore make it difficult to collect data from different sources and studies and integrate them for comprehensive analysis. DO resolves this issue and helps to not only harmonize inconsistent terms across data sites but also to list and connect these terms in their clinical vocabulary cross references (OMIM, NCIt, UMLS, MeSH, SNOMED_CT and ICD). Second, DO provides a precise mapping between all of the cancer terms selected from the multiple cancer databases. Queries of terms in DO return the corresponding DOIDs and DO terms. Third, DOID provides a machine readable ID entry in a hierarchical structure. This hierarchical structure allows terms to be tracked back to their upper level (parent) or lower level (child) nodes if necessary. Furthermore, DO’s TopNodes_ DOcancerslim provides a trimmed down version of DO that can be used for data integration and pan-cancer analysis that involves data integration from multiple sources (17, 25). If all data are mapped to DO terms then users can query the data, e.g. to identify SNVs that are present in the top-level node ovarian cancer (DOID:2394) or a specific child term of ovarian cancer such as ovarian endometrial cancer (DOID:6212).

## Conclusion

Overall, 386 cancer terms were collected from multiple cancer databases and were mapped to 187 DO cancer terms. This subset of DO terms were mapped to a group of 63 DO upper level cancer types and saved to the TOPNodes_DOcancerslim.obo file. Future plans include expanding the set of terms and also updating the DO to cancer term source mappings at least once a year and developing better dissemination and community input collection methods. This is an ongoing project and we expect to attract additional participants to contribute to either mapping of cancer terms to DO or requesting for new terms to be added. The overarching goal of the DO cancer project is to provide a comprehensive set of hierarchical DO terms which allow granular mapping of the cancer disease terms from multiple projects.

## Supplementary Data

Supplementary data are available at *Database* Online.

Supplementary Data
