# Supplementary material for: Generating a focused view of disease ontology cancer terms for pan-cancer data integration and analysis
Source: Database (Oxford). 2015 Apr 4;2015:bav032. doi: 10.1093/database/bav032 (PMC4385274; doi:10.1093/database/bav032)
Supplement: Supplementary Data [file supp_2015_bav032_index.html]

Generating a focused view of disease ontology cancer terms for pan-cancer data integration and analysis — Supplementary Data 

# Generating a focused view of disease ontology cancer terms for pan-cancer data integration and analysis

## Supplementary Data

files

**Files in this Data Supplement:**

- Supplementary Data - xlsx file
